# Observations of boundary layer wind and turbulence of a landfalling tropical cyclone

**DOI:** 10.1038/s41598-022-14929-w

**Published:** 2022-06-30

**Authors:** Zhongkuo Zhao, Ruiquan Gao, Jun A. Zhang, Yong Zhu, Chunxia Liu, P. W. Chan, Qilin Wan

**Affiliations:** 1 Guangzhou Institute of Tropical and Marine Meteorology/Guangdong Provincial Key Laboratory of Regional Numerical Weather Prediction, China Meteorological Administration, GuangDong, 510640 China; 2 Meteorological Bureau of Shenzhen Municipality, GuangDong, 518040 China; 3 NOAA/AOML/Hurricane Research Division, University of Miami/CIMAS, Miami, FL 33149 USA; 4 Hong Kong Observatory, Hong Kong, 999077 China

**Keywords:** Atmospheric science, Atmospheric dynamics

## Abstract

This study investigates the atmospheric boundary layer structure based on multiple-level tower observations with a height of 350 m during the landfall of Super Typhoon Mangkhut (2018). Results show a layer of log wind profile outside of the radius of maximum wind speed with a height of 100 m or larger. The log layer height increases with the wind speed. The height of the constant flux layer reaches ~ 300 m for 10-m wind speeds less than 13 m s^−1^ while this height decreases with the wind speed. Momentum fluxes and turbulent kinetic energy increase with the wind speed at all vertical levels. The drag coefficient and surface roughness length estimated at the tower location have values of 7.3 × 10^–3^ and 0.09 m, respectively, which are independent of wind speed. The estimated vertical eddy diffusivity and mixing length increase with height up to ~ 160 m and then slowly decrease with height. The vertical eddy diffusivity increases with the wind speed while the vertical mixing length has no dependence on the wind speed. Comparing our results with previous work indicates that the vertical eddy diffusivity is larger over land than over ocean at a given wind speed range.

## Introduction

Physical processes in the planetary boundary layer (PBL) have been known to play an important role in the intensity change of a tropical cyclone (TC)^1–5^. Previous numerical studies have shown that the simulated TC intensity and structure are very sensitive to turbulence parameterization methods used in the PBL schemes^6–9^. This sensitivity has also been confirmed in the operational TC forecasts by the Hurricane Weather and Research Forecast (HWRF) model. Observation based modifications of the vertical and horizontal turbulent mixing strengths have led to substantial improvements in HWRF’s intensity forecast skills over ocean^10–14^. Of note, the available flux observations have been limited to either the outer core region (R > 100 km) or near the top of the PBL in the eyewall region^15–17^.

The magnitudes of surface roughness lengths over land are greater than those over ocean by 1–2 orders^18^, and this roughness enhancement induces more turbulent mixing in the PBL over land^19^. Previous studies have demonstrated that increasing the vertical eddy diffusivity over land in the PBL scheme of HWRF improved forecasts of track, rainfall, storm size, and wind structure of several landfalling storms^19^. However, turbulence observations in landfalling TCs were mainly limited to the near surface layer (z < 10 m)^20,21^. Until now, no previous studies have shown multi-level flux data above 100 m. On the other hand, more observational studies have investigated the mean PBL structure in landfalling storms especially using Doppler radar data with a focus on structural transition from ocean to land^22–24^. The interaction of mean and turbulence structures remains to be investigated further.

To fill the gap in understanding turbulent mixing processes in landfalling TCs, the present study presents turbulence observations by a multilevel high tower in Typhoon Mangkhut (2018). Turbulence parameters such as turbulent kinetic energy (TKE), momentum flux, vertical eddy diffusivity and mixing length are estimated using fast-response wind data. Multilevel wind observations are used to document the mean kinematic structure including the wind shear and provide an independent estimate of surface fluxes. The objective is to document turbulent characteristics of the low-level PBL and its potential linkage to the mean kinematic structure over land.

## Data and methodology

Typhoon Mangkhut formed in the northwest Pacific Ocean on 7 September 2018. At 09:00 UTC on 16 September, it made landfall at the coastal area of Jiangmen District, Guangdong Province of China, as a super typhoon with the maximum 10-min wind speed of 45 m s^−1^. Wind speeds of > 33 m s^−1^ were recorded by a local weather station in the Guangdong Province with a period of > 13 h.

**Figure 1:**
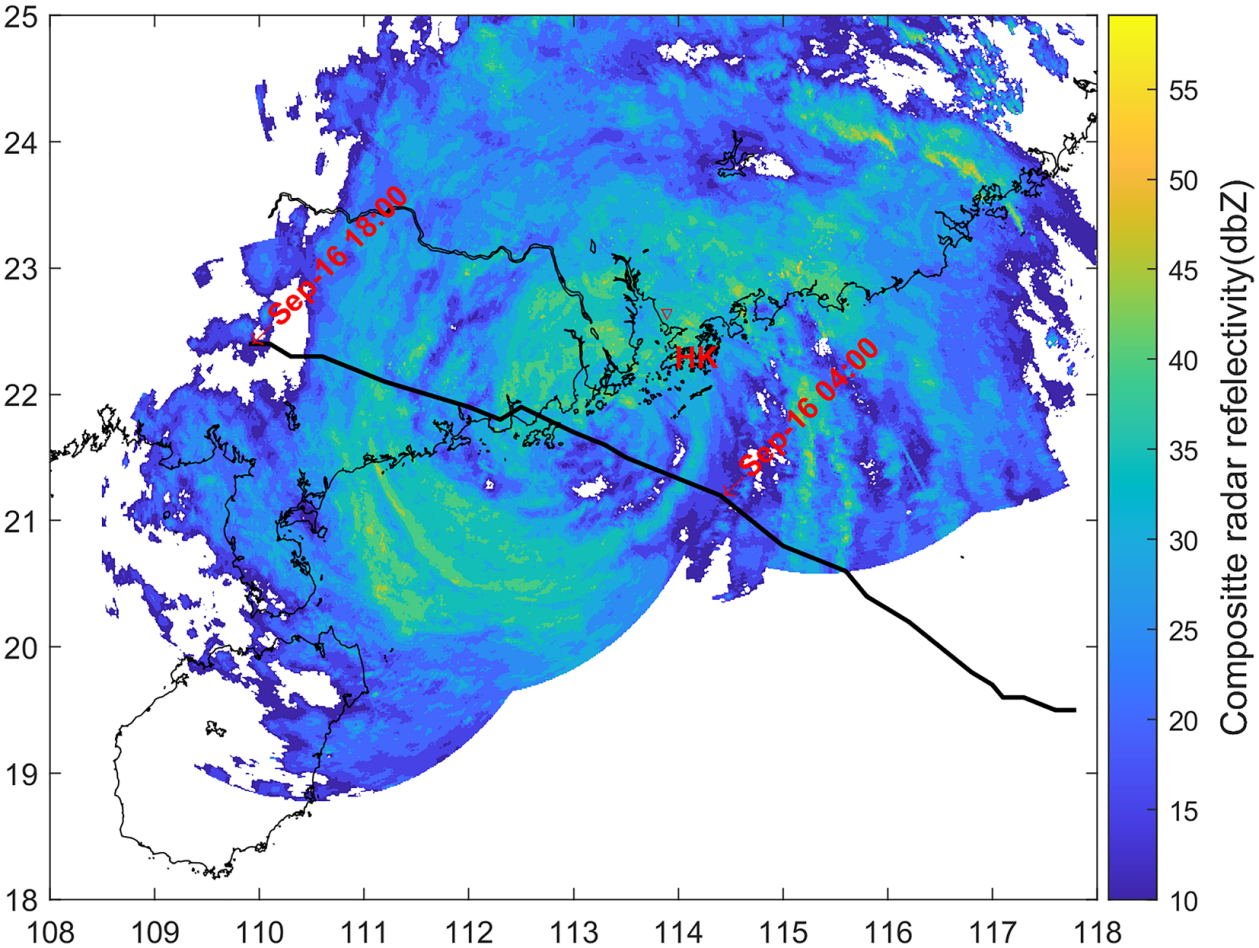
Plots of radar reflectivity composite at 07:00 UTC on September 16 when the tower was closest to the typhoon center, and real-time track (bold solid line) of Typhoon Mangkhut (1822) in the coastal area of South China from 14:00 UTC on September 15, 2018, to 18:00 UTC on September 16, 2018. The storm track data was obtained from the National Meteorological Centre of China. The circle and x symbols denote the location of the Shenzhen Meteorological Tower. HK represents Hongkong.

**Figure 2:**
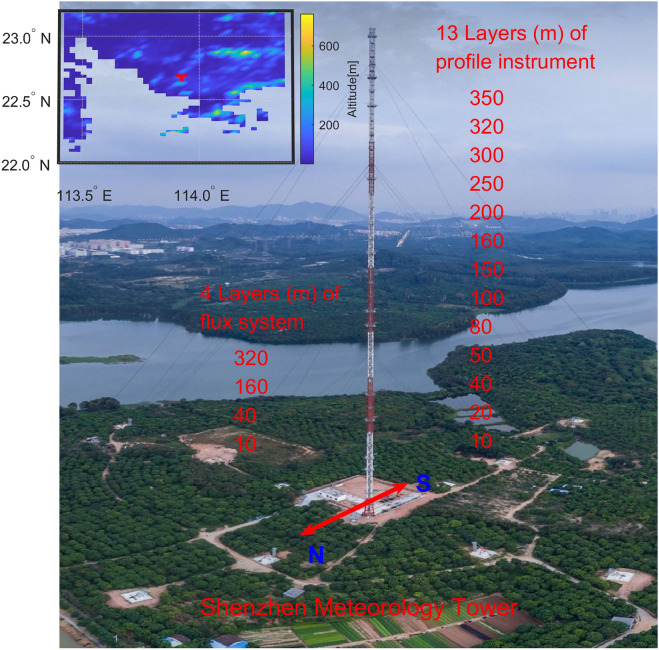
Instrument layout in the Shenzhen Meteorological Tower (SMT). The heights of the devices for measuring vertical wind profiles and turbulent fluxes are labelled. The water behind the SMT is a reservoir. The upper-left subpanel shows the local topography, and the red marker (T) denotes the SMT.

Figure 1 shows Mangkhut’s track from 14:00 UTC on 15 September to 18:00 UTC on 16 September 2018, when the maximum 10-min average surface wind of the storm was > 33 m s^−1^. The composite radar reflectivity in Fig. 1 was a snapshot at 07:00 UTC on 16 September. The radius of maximum winds (RMW) of Mangkhut was reported to be 105 km when the storm approached Hong Kong^25^. Data from the Shenzhen Meteorological Tower (referred to as SMT hereafter, Fig. 2) were analysed in this study. The location of the SMT is also shown in Fig. 1. The fetch over land at the SMT is > 40 km, although the distance from the SMT to the nearest coastline is ~ 9 km. Previous studies^24^ have shown that the transition of the TCBL across the coastal region mainly occurred within first 5 km from the coastline over land. The terrain type surrounding the SMT is tropical shrub.

The SMT contains a total of 13 levels of slow-response (1 Hz) wind observations including 4 levels of fast-response (10 Hz) wind data for eddy-covariance flux calculations. The heights for slow-response wind measurements are 10, 20, 40, 50, 80, 100, 150, 160, 200, 250, 300, 320 and 350 m. Flux measurement systems are located at 10, 40, 160, and 320 m, respectively. Three observation systems (Vaisala anemometer WAA15, wind vane WAV15 and Vaisala ultrasonic wind sensor WMT703) were used to measure wind velocities for calibration and quality control purposes. The data were not used when wind observations from the three instruments were significantly different.

The magnitude of momentum flux _(τ)_ was calculated using the standard eddy-covariance method in the following form:

1 $$\tau = \rho \left| { - \overline{{w^{^{\prime}} u^{^{\prime}} }} \hat{i} - - \overline{{w^{^{\prime}} v^{^{\prime}} }} \hat{j}} \right|,$$

where _ρ_ is the air density, _u′, v′, and w′_ are turbulent fluctuations of the zonal, meridional, and vertical components of wind velocities, respectively, and the overbar denotes average of a 30-min period of continuous wind observations that passed a stationary test^20^.

**Figure 3:**
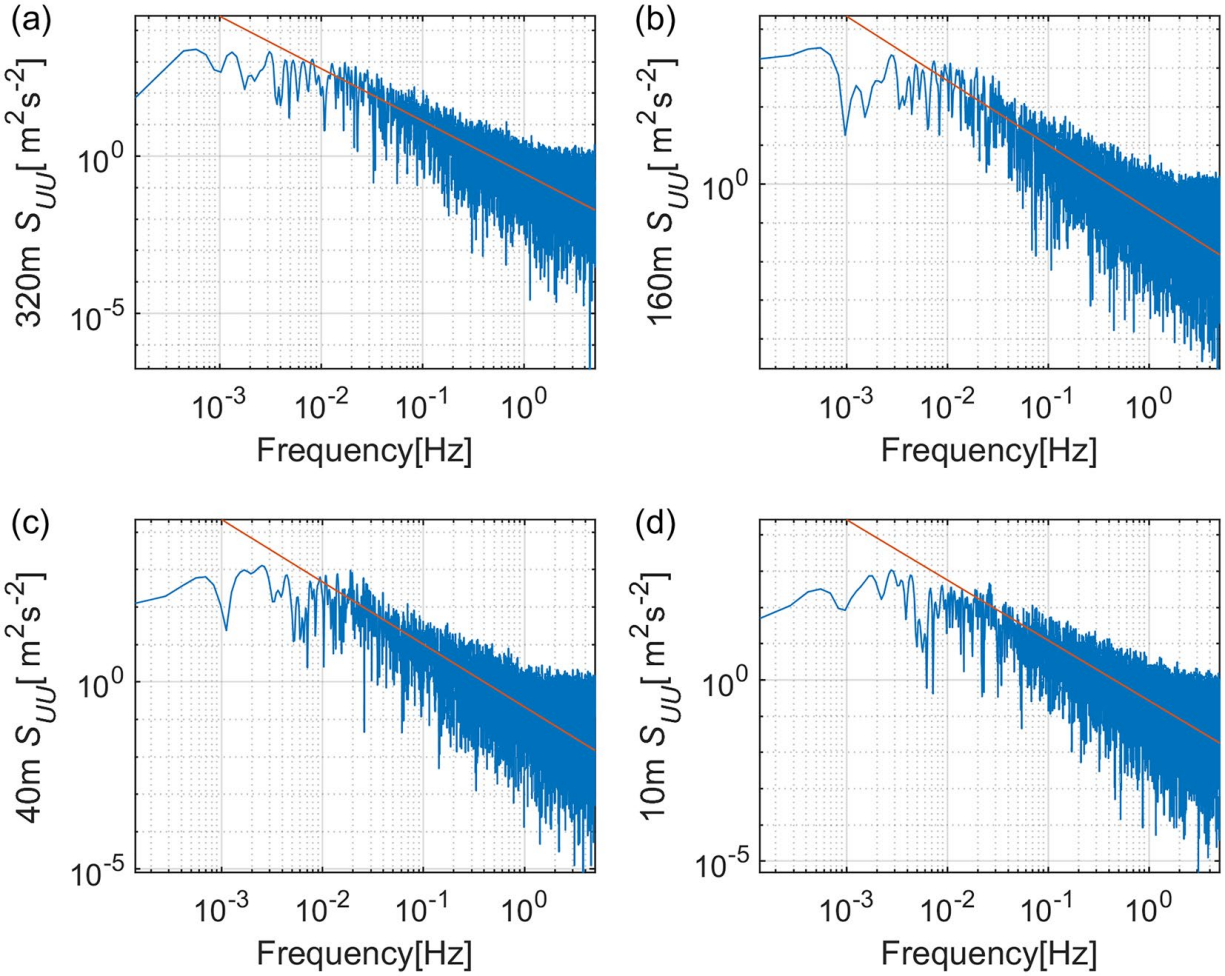
Examples of along-wind velocity spectra at (**a**) 320 m, (**b**) 160 m, (**c**) 40 m and (**d**) 10 m height. The red lines with a slope − 5/3 indicate the inertial subrange. The record time is 2018-09-16 07:30.

**Figure 4:**
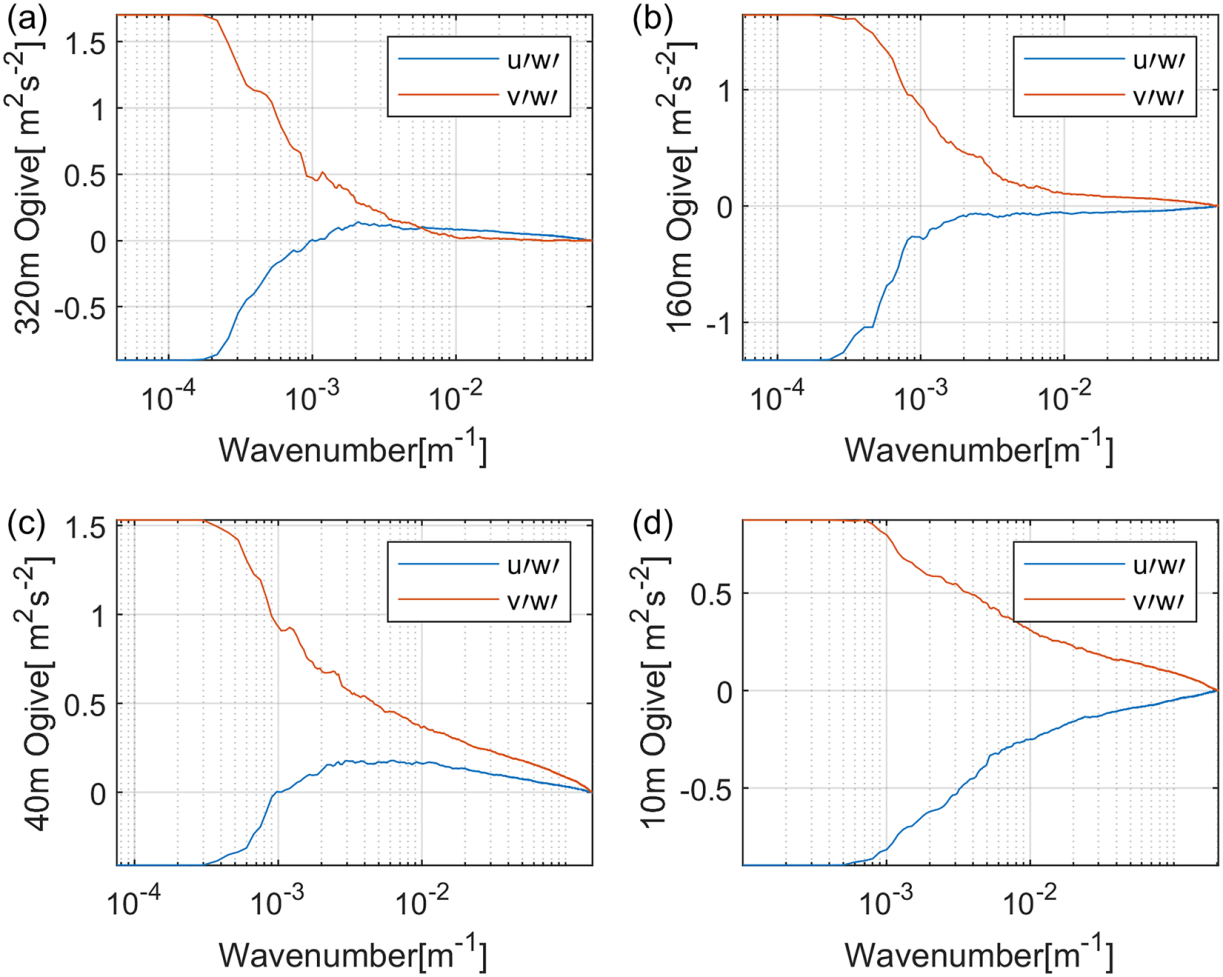
Cumulative sum of cospectrum (Ogive) curves of the two components of momentum fluxes verse the horizontal wavenumber at height (**a**) 320 m, (**b**) 160 m, (**c**) 40 m, and (**d**) 10 m. The record time is 2018-09-16 07:30.

Velocity spectra, cospectra and their cumulative sums (ogives) were checked to select flux legs^18^. Flux estimates are considered reliable when the wind spectral curve plotted against the frequency in a logarithmic scale has a slope of − 5/3 in the inertial sub-range, with examples shown in Fig. 3. In addition, the flatness of the ogive curve of the cospectrum at both the low and high frequency ends is required for stationarity requirement (Fig. 4).

The vertical eddy diffusivity (*K*_m_) is estimated using the momentum flux and strain rate in the form of:

2 $${K}_{m}=\tau /(\rho S),$$

Here, the strain rate *S*is calculated as

3 $$S = \sqrt {\left( {\frac{{\partial \overline{u}}}{\partial z}} \right)^{2} + \left( {\frac{{\partial \overline{v}}}{\partial z}} \right)^{2} } .$$

The same method as used by Zhang and Drennan^15^ was followed to compute *S*. The weighted linear least square method was used to smooth the vertical profiles of u and v components before calculating the vertical gradients and strain rate. The vertical mixing length (*l*) is then estimated using the eddy diffusivity and strain rate,

4 $$l = \sqrt {\frac{{K_{m} }}{l}} .$$

To estimate $K_{m}$, the non-local Medium Range Forecast (MRF) PBL scheme^26^ was modified based on observations over ocean in HWRF^11,19^ using a tuning parameter α in the form of

5 $$K_{m} = kzu_{*} \left[ {\alpha (1 - z/h)^{2} } \right]$$

where κ = 0.4 is the von Karman constant, *z* is height, u_*_ is the friction velocity, and *h* is the PBL height of the boundary layer. How this parameter may be tuned over land will be discussed later. The mixing length is usually formulated by *kz* and an asymptotic mixing length *l*_∞_ (Blackadar scheme^27^) in the form of

6 $$\frac{1}{l} = \frac{1}{kz} + \frac{1}{{l_{\infty } }}.$$

This formulation limits the mixing length to *kz* when approaching the surface. In the present study, we fit the observational data using both Eq. (6) and the following equation

7 $$\frac{1}{l} = \frac{1}{z} + \frac{1}{{l_{\infty } }},$$

which implies increasing *l* with *z* close to the surface.

## Results

**Figure 5:**
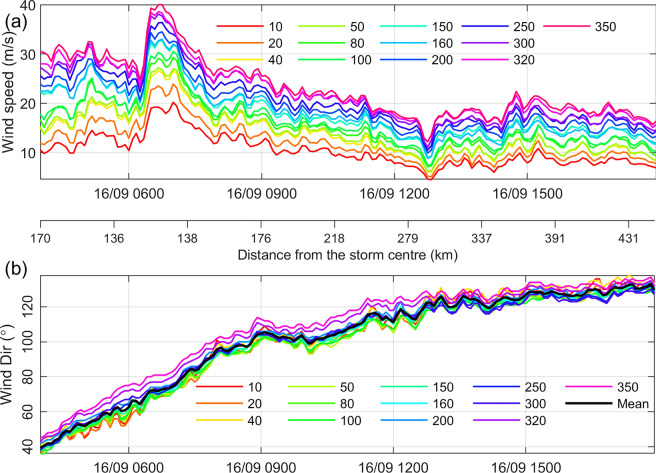
Observations obtained at 13 vertical levels of the Shenzhen Meteorological Tower from 04:00 to 18:00 on 16 September 2018: (**a**) 10-min average wind speed and (**b**) 10-min average wind direction. In panel (**b**), the thick black line shows the mean wind direction averaged over the 13 levels. The axis between the two panels shows the relative distance of SMT from the storm centre (km) according to the real-time track data from China Meteorology Administration.

Figure 5 shows the 10-min averaged wind speed and direction at 13 levels of the SMT from 04:00 UTC to 18:00 UTC on 16 September 2018, when the distance from the SMT to the storm center was approximately 130–200 km. The strongest wind was captured at ~ 07:00 UTC due to the passage of the eyewall. The wind speed increases with height as expected (Fig. 5a). In general, the wind vector rotates clockwise by ~ 20° over the height of the tower (Fig. 5b). The degree of wind direction variation with height substantially decreased after 13:00 UTC.

**Figure 6:**
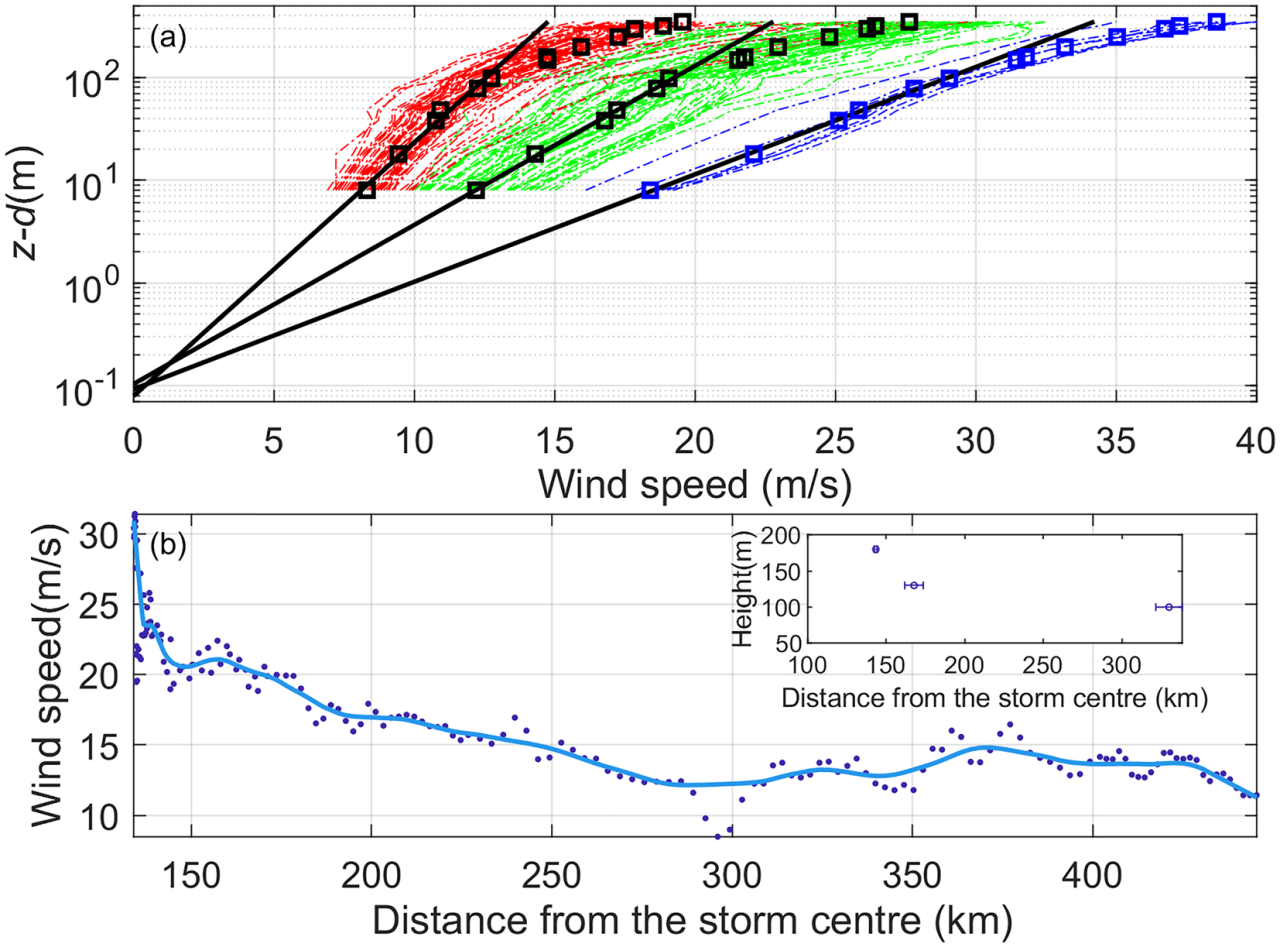
(**a**) Vertical profiles of 10-min average wind speed. Each symbol shows the mean value at a given vertical level. The 3 fitted lines are based on the data below 100 m using the least square method. The wind speed data are grouped according to 3 wind speed ranges: 5–10 m/s (red), 10–15 m/s (green) and 15–20 m/s (blue). (**b**) Multiple-level mean wind speed (dot) and the fitting line (solid blue line) as a function distance from the TC center. The subpanel in (**b**) shows the dependence of the logarithmic surface layer height on the distance from TC center. The horizontal bars indicate the standard deviations of the distances grouped into 3 bins according to the 10-m wind speed ranges, as in panel (**a**).

Vertical wind profiles are plotted as a function of height with a logarithmic scale in Fig. 6a. Bin averages of 3 wind speed groups (5–10 ms^−1^, 10–15 ms^−1^ and 15–20 ms^−1^) are shown at all levels. The mean wind profile follows the log relationship with height below 100 m height as illustrated by the best fit black line for the three groups. The existence of a logarithmic wind profile in the surface layer is a basic assumption in modeling the TCBL^28^ which was supported by observations^29^. Of note, Smith and Montgomery^30^ questioned the existence of a logarithmic layer in the TC inner core region, where the strong radial gradient of a TC primary circulation (i.e., tangential wind) may invalidate the homogeneous assumption in deriving the log layer. However, our data provided a firm support of the existence of a logarithmic wind profile in the surface layer of a storm at a radius of ~ 1.3 times RMW.

The lowest 100 m bin-averaged wind speeds in Fig. 6a are fitted as a function of height using a least-square linear method and equation

8 $$U_{z} = \frac{{u_{*} }}{k}\ln \left( {\frac{z - d}{{z_{0} }}} \right),$$

where *U*_*z*_ is the mean wind speed at height *z*, *z*_0_ is the roughness length, and *d* is the zero-velocity displacement distance set to be 2 m as 2/3 of the height of surrounding shrubs which is ~ 3 m. The intercept and slope of the fitted line produce a measure of *z*_0_ and $u_{*} /k$, respectively. The drag coefficient (*C*_*D*_) is then obtained by $C_{D} = (u_{*} /U_{10} )^{2}$. Values of $u_{*}$, *z*_0_ and *C*_*D*_ are summarized in Table 1. *z*_0_ and *C*_*D*_ are independent of *U*_*10*_, and have mean values of 0.09 m and 0.0073, respectively. This behaviour of nearly constant *z*_0_ and *C*_*D*_ is as expected over land following the surface layer theory^31^.

**Table 1:** Estimates of friction velocity (u_*_) and aerodynamic roughness length (*z*_0_) obtain by a log wind profile fit.

*U*_*10*_ (m/s)	u_*_ (m/s)	*z*_0_ (m)	1000 × *C*_*D*_
8.5	0.70	0.08	6.9
12.8	1.12	0.10	7.7
19.4	1.66	0.09	7.3

It is also noticed in Fig. 6a that the larger the wind speed range, at a higher level the mean wind speed is closer to the best fit line. This feature indicates that the surface layer defined as the layer with a logarithmic wind profile deepens with the wind speed. The surface layer heights are estimated to be 100, 130 and 180 m for 5–10 ms^−1^, 10–15 ms^−1^ and 15–20 ms^−1^ groups, respectively. Another interesting feature in these wind profiles is that a log wind profile exists even above 100 m height, but with a smaller slope at higher wind speeds. In addition, the wind speed increases more quickly with height above the log layer than below for all three groups. The characteristics of the wind profiles are consistent with those of an internal boundary layer (IBL) that forms due to the surface roughness change from rough to smooth^18^. Of note, in the absence of mesoscale effects, the TC boundary layer is typically considered statically neutral due to strong wind shear^28^. The IBL height (*h*_I_) is defined as:

9 $$h_{I} = cZ_{0D} \left( {\frac{X}{{Z_{0D} }}} \right)^{0.8} ,$$

where $Z_{0D}$ is the downstream roughness length, and *c* is a stability constant with a value of 0.35^32^, *X* is the distance to the location of upstream surface roughness change. Using $Z_{0D} = 0.09 m$, and setting the internal boundary layer height as 100 m, gives *X* of 2.1 km. The change in slope of the wind profile above the log layer is different from that over ocean as documented by Vickery et al.^33^, which is likely due to the surface roughness differences and no formation of IBL over ocean.

Figure 6b shows that the mean wind speed increases with the decreasing distance from the storm center as expected. This result in combination with Fig. 6a indicate that the layer of log wind profile deepens toward the storm center outside of the RMW. The logarithmic surface layer heights of these three groups are shown in the subset plot of Fig. 6b. Note that the mean distances for 5–10 ms^−1^, 10–15 ms^−1^ and 15–20 ms^−1^ groups are 3.14, 1.60 and 1.36 times the RMW, respectively.

**Figure 7:**
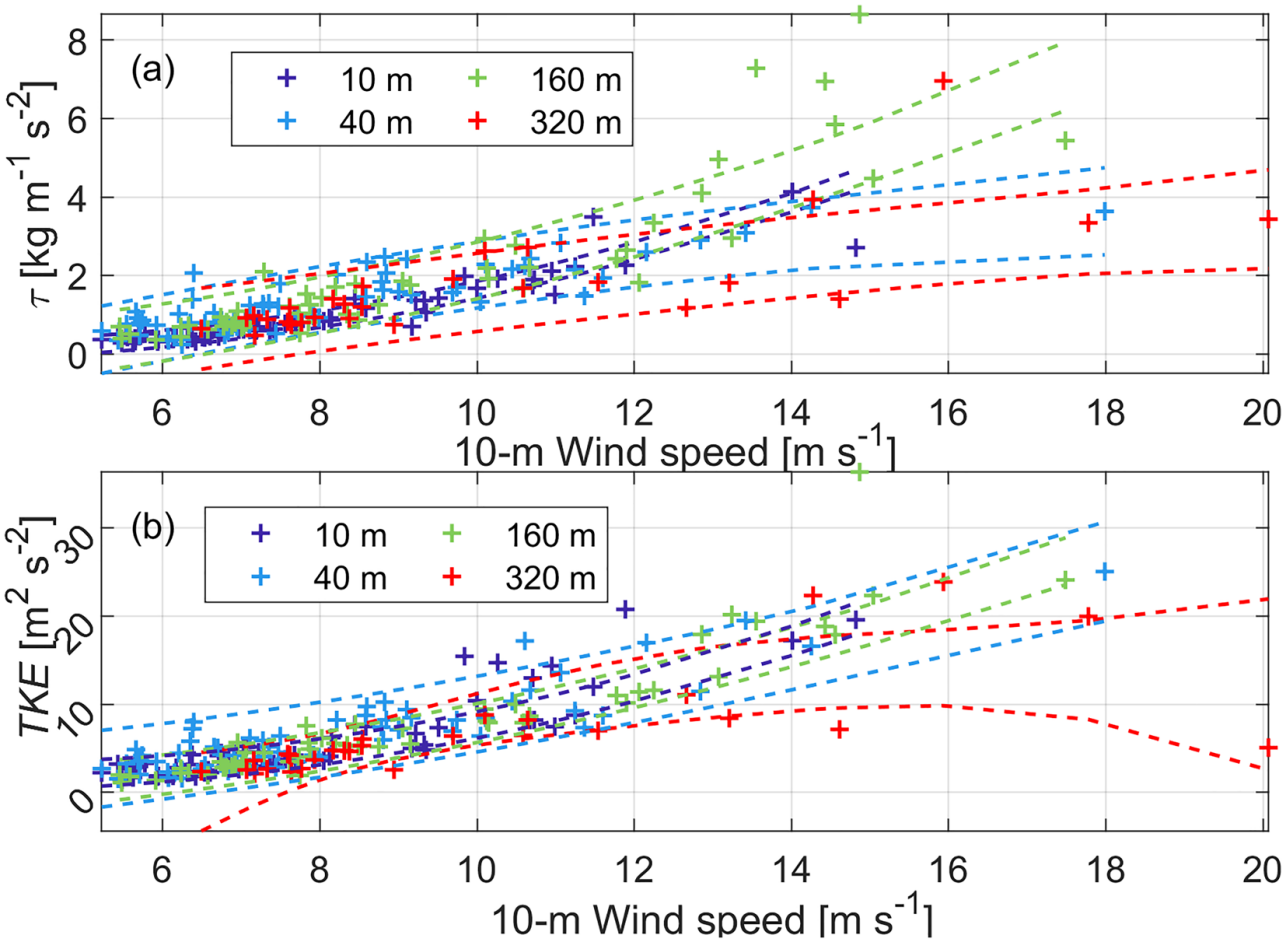
Plots of (**a**) momentum flux (τ) and (**b**) turbulent kinetic energy ($TKE = (\overline{{u^{^{\prime}2} }} + \overline{{v^{^{\prime}2} }} + \overline{{w^{^{\prime}2} }} )/2$) as a function of the 10-m wind speed at four levels, 10, 40, 160 and 320 m. The 4 couples of dashed lines in same color as the + in (**a**) and (**b**) denote the corresponding 95% confidence intervals. Panels (**a**) and (**b**) share the same legends.

Figure 7a shows the momentum fluxes at 4 measurement levels (i.e., 10, 40, 160 and 320 m) as a function of 10-m wind speed, indicating an increasing trend of the momentum flux with the wind speed. The values of momentum fluxes at 10, 40 and 160 m heights are close to each other as indicated by the overlapping 95% confidence intervals of these groups at all available wind speeds. On the other hand, the 95% confidence interval of the 320 m height group overlaps with all other three groups at relatively low wind speed ranges. This result suggests that 320 m was above the surface layer defined by the constant flux layer at wind speeds > 13 ms^−1^ while 160 m is within the surface layer during the landfall of Typhoon Mangkhut.

Figure 7b shows the turbulent kinetic energy ($TKE = (\overline{{u^{^{\prime}2} }} + \overline{{v^{^{\prime}2} }} + \overline{{w^{^{\prime}2} }} )/2$) at the 4 levels of flux observation as a function of the 10-m wind speed. The TKE at each level increases with the wind speed (with a mean correlation coefficient of 0.85). The TKE values at wind speeds > 10 m/s are comparable to those in large eddy simulations of a landfall hurricane given by Zhu^34^ as well as those based on flight-level and Doppler radar observations^22,35^. Of note, there is no statistically significant difference between the TKE at these levels for a given wind speed according to their overlapped 95% confidence intervals.

**Figure 8:**
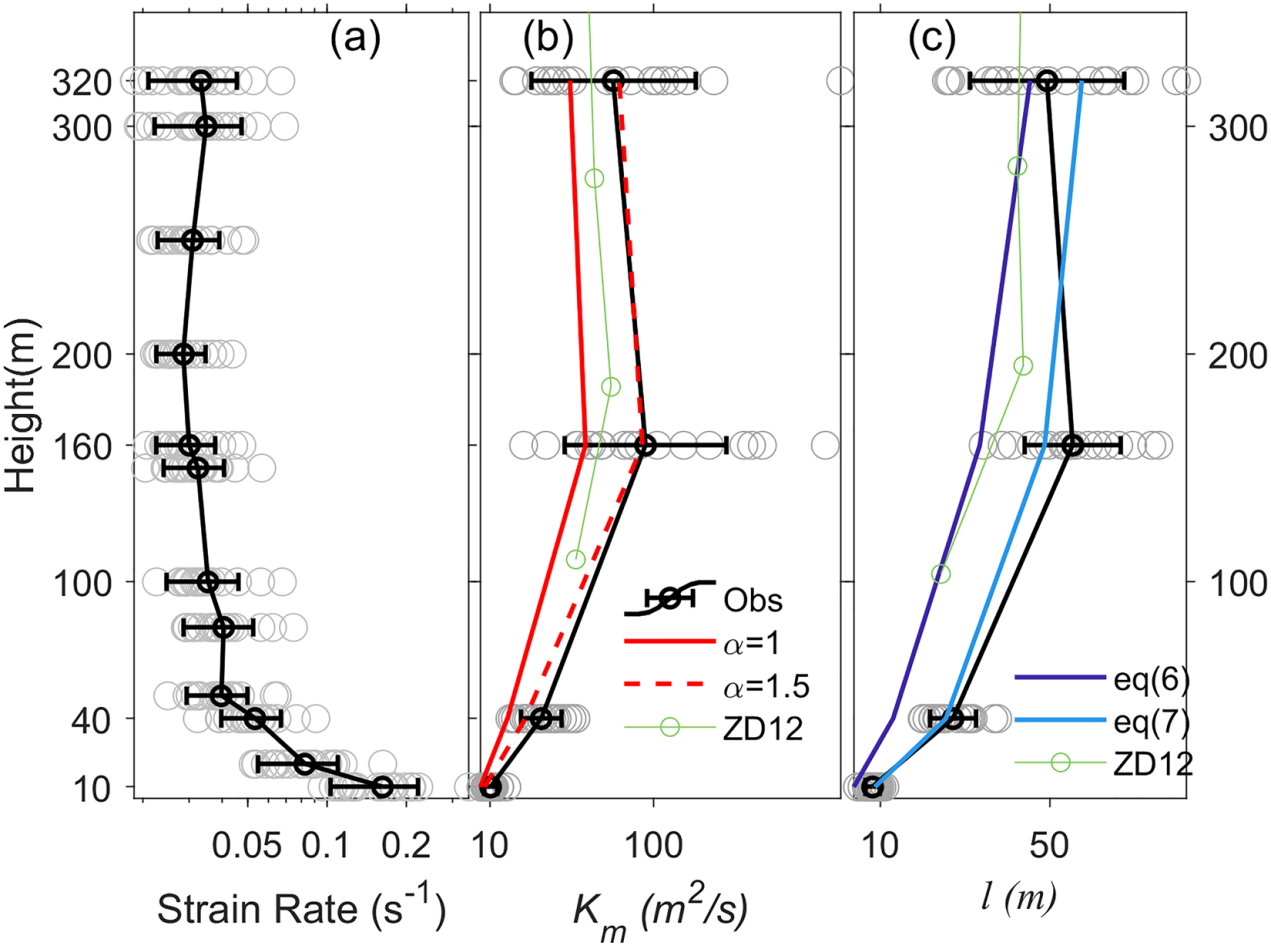
Vertical profiles of (**a**) strain rate, (**b**) vertical eddy diffusivity *K*_*m*_, and (**c**) vertical mixing length *l*. The average values and standard deviations (error bars) are also shown. Panels (**b**) and (**c**) also show the best fit lines. ZD12 denotes Zhang and Drennan (2012).

The strain rate is plotted against the height in logarithmic scale in Fig. 8a. The maximum strain rate appears at 10 m altitude with an average value of 0.16 s^−1^. The strain rate decreases with height up to ~ 200 m before levelling off. The local minimum mean value is 0.029 s^−1^ at 200 m height. This behaviour of vertical variation of strain rate is similar to that over the ocean^15^. However, the values of the strain rate in our study are much greater than those over ocean for similar wind speeds, which is attributed to the larger surface roughness length over land than over ocean.

Vertical profiles of the estimated vertical eddy diffusivity (*K*_*m*_) show an increasing trend with height below 160 m but a decreasing trend above (Fig. 8b). The mean values of *K*_*m*_ are 11 and 38 m^2^ s^−1^ at 10 m and 40 m height, respectively. It peaks at 160 m with an average value of 96 m^2^ s^−1^. At 320 m, the mean value of *K*_*m*_ decreases to 78 m^2^ s^−1^. The trend of variation of *K*_m_ with height here is similar to that over the ocean^15^, where a maximum *K*_m_ is located at approximately 190 m height. However, our values of *K*_m_ are 50% greater than those over ocean^15^ at an equivalent height and wind speed range. The enhanced *K*_m_ over land is mainly due to the larger roughness length indicting stronger vertical mixing than over ocean.

According to Eq. (2), within a constant momentum flux layer, *K*_m_ should have a maximum at the height of minimum strain rate. The minimum strain rate is at ~ 200 m altitude as shown in Fig. 8a. Thus, it is speculated that the maximum of *K*_m_ appears close to this height if the constant flux layer extends above 200 m. In the *K-*profile parameterization scheme as in Eq. (5), *K*_m_ maximizes at ~ 1/3 of the PBL height (*h*). Setting *α* = 1.5 in Eq. (5) and using *h* = 675 m gives values of *K*_m_ close to our observations, again suggesting *K*_m_ is larger over land than over ocean.

The vertical mixing length (*l*) is estimated using Eqs. (2)–(4) and shown in Fig. 8c. The mean values of *K*_m_ at 10 and 40 m altitudes are 8 m and 27 m, respectively. Note that Tang et al.^20^ reported values of *l* being 10—20 m at 27 and 42 m altitudes near the coastline. The average values of *l* at 160 m and 320 m altitudes in our study are 55 m and 49 m, respectively, which are greater than those (~ 40 m) over ocean^15^. If the vertical profile of *l* is fitted according to the Blackadar scheme (Eq. 6), a value of 104 m is obtained for *l*_∞_, while a value of 67 m is obtained for *l*_∞_ when using Eq. (7). The shape of the *l* profile is much closer to that of Eq. (7) than Eq. (6) as shown in Fig. 8c. The root-mean-square-error of the best fit using Eq. (6) is nearly twice of that using Eq. (7) compared to observed values.

**Figure 9:**
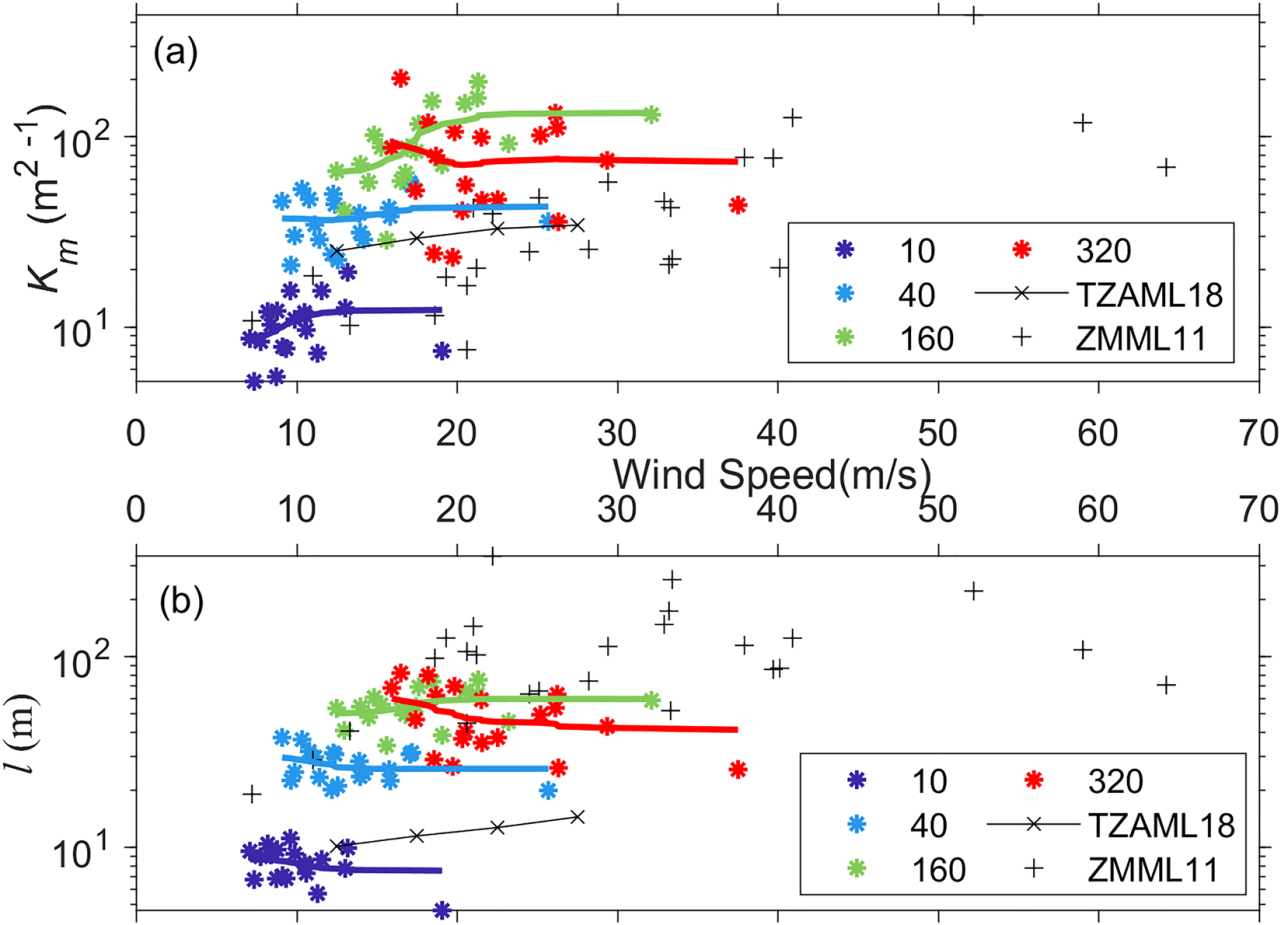
Plots of (**a**) vertical eddy diffusivity (K_m_) and (**b**) vertical mixing length, at 4 vertical levels, as a function of the wind speed. TZAML18 stands for Tang et al. (2018), and ZMML11 stands for Zhang et al. (2011a). In (**a**) and (**b**), the best fit line at each level is also shown.

Figure 9a shows *K*_m_ at each measurement level as a function of wind speed. Besides the height dependence shown in Fig. 8b, *K*_m_ also has a wind speed dependence. At 10 m and 160 m altitudes, *K*_m_ increases with the mean wind speed up to 13 m s^−1^ and 21 m s^−1^, respectively. However, at 40 m and 320 m altitudes, the dependence of *K*_m_ on the wind speed is relatively small. This increasing trend of *K*_m_ with the wind speed in our observations generally agrees with previous studies^16,20^. The dependence of *l* on the wind speed at each level is shown in Fig. 9b, indicating that there is no significant relationship between *l* and the wind speed. Of note, the result of Tang et al.^20^ showed a weak dependence of *l* on the wind speed, while Zhang et al.^16^ result showed no dependence of *l* on the wind speed in agreement with our result.

## Discussions and conclusions

In this study, multi-level tower observations of the low-level boundary layer structure of a landfalling TC were presented. Both mean and turbulence structures of the boundary layer were investigated. The mean wind profiles showed that the depth of the log wind profile layer was close to 100 m for the mean 10-m wind speed of 8.3 m s^−1^. This log layer depth increased to ~ 200 m as the mean 10-m mean wind speed increased to 18 m s^−1^. The eddy-covariance momentum flux data showed that 300 m was within the constant flux layer for 10-m wind speeds < 13 m s^−1^ while 300 m was beyond the constant flux layer at higher wind speeds. Our result suggests that the top of the log wind profile layer may not represent the surface layer depth defined by a constant flux layer. Furthermore, this result indicates that the surface layer depth defined using the constant flux layer method decreases with the increasing wind speed. Dropsonde composites over ocean also showed the PBL height decreased toward the storm center outside the RMW. Asassuming that the surface layer height variation trend with radius follows that of the boundary layer height, our result agrees with the previous finding based on dropsonde composites. This assumption requires evaluation in the future when collocated high-resolution Doppler radar or Doppler profile observations with flux observations are available.

Turbulence parameters, including the momentum flux, TKE, drag coefficient, roughness length, strain rate, vertical mixing length, and vertical eddy diffusivity were estimated using the tower data. The dependence of these parameters on height and wind speed were examined. The drag coefficient and roughness length were nearly constant with values of 0.0073 and 0.09 m, respectively. The vertical eddy diffusivity generally increased with the wind speed. The dependence of the mixing length on wind speed was very weak at all levels. The mean values of *l* were 8 m, 27 m, 55 m and 49 m, while those of *K*_m_ were 11, 38, 96 and 78 m^2^ s^−2^ at 10 m, 40 m, 160 m and 320 m levels, respectively.

*K*_m_ increased with height up to about 160 m altitude and then weakly decreased with height. The vertical mixing length increased with height up to 160 m and then became nearly constant above this height. The nonlocal MRF scheme and the Blackadar mixing length scheme were evaluated using our observational estimates. Results showed that an enhancement factor is needed to improve both the original MRF and Blackdar schemes over land. Our result showed that the vertical mixing length may be parameterized as a function of *z* rather than the usual *l *≈ *kz* relationship. Future field experiments are required to further study the relationship between the mixing length and height during landfalling TCs, especially at wind speed range higher than that reported by the present study.

## References

[1] Smith RK, Zhang JA, Montgomery MT (2017). The dynamics of intensification in a Hurricane Weather Research and Forecasting simulation of Hurricane Earl. Q. J. R. Meteorol. Soc..

[2] Zhang JA, Rogers RF (2019). Effects of parameterized boundary layer structure on hurricane rapid intensification in shear. Mon. Weather Rev..

[3] Kilroy G, Montgomery MT, Smith RK (2017). The role of boundary-layer friction on tropical cyclogenesis and subsequent intensification. Q. J. R. Meteorol. Soc..

[4] Riemer M, Montgomery MT, Nicholls ME (2010). A new paradigm for intensity modification of tropical cyclones: Thermodynamic impact of vertical wind shear on the inflow layer. Atmos. Chem. Phys..

[5] Cione JJ, Kalina EA, Zhang JA, Uhlhorn EW (2013). Observations of air–sea interaction and intensity change in hurricanes. Mon. Weather Rev..

[6] Braun SA, Tao W-K (2000). Sensitivity of high-resolution simulations of Hurricane Bob (1991) to planetary boundary layer parameterizations. Mon. Weather Rev..

[7] Nolan DS, Stern DP, Zhang JA (2009). Evaluation of planetary boundary layer parameterizations in tropical cyclones by comparison of in situ observations and high-resolution simulations of hurricane Isabel. Part II: Inner-core boundary layer and eyewall structure. Mon. Weather Rev..

[8] Smith RK, Thomsen GL (2010). Dependence of tropical-cyclone intensification on the boundary-layer representation in a numerical model. Q. J. R. Meteorol. Soc..

[9] Chen X, Bryan GH, Zhang JA, Cione JJ, Marks FD (2021). A framework for simulating the tropical cyclone boundary layer using large-eddy simulation and its use in evaluating PBL parameterizations. J. Atmos. Sci..

[10] Gopalakrishnan SG (2013). A study of the impacts of vertical diffusion on the structure and intensity of the tropical cyclones using the high-resolution HWRF system. J. Atmos. Sci..

[11] Zhang JA, Nolan DS, Rogers RF, Tallapragada V (2015). Evaluating the impact of improvements in the boundary layer parameterization on hurricane intensity and structure forecasts in HWRF. Mon. Weather Rev..

[12] Zhang JA, Rogers RF, Tallapragada V (2017). Impact of parameterized boundary layer structure on tropical cyclone rapid intensification forecasts in HWRF. Mon. Weather Rev..

[13] Hazelton A, Zhang JA, Gopalakrishnan S (2022). Comparison of the performance of the observation-based hybrid EDMF and EDMF-TKE PBL schemes in 2020 tropical cyclone forecasts from the global-nested hurricane analysis and forecast system. Weather Forecast..

[14] Zhang JA (2018). Evaluating the impact of improvement in the horizontal diffusion parameterization on hurricane prediction in the operational Hurricane Weather Research and Forecast (HWRF) Model. Weather Forecast..

[15] Zhang JA, Drennan WM (2012). An observational study of vertical eddy diffusivity in the hurricane boundary layer. J. Atmos. Sci..

[16] Zhang JA, Marks FD, Montgomery MT, Lorsolo S (2011). An estimation of turbulent characteristics in the low-level region of intense hurricanes Allen (1980) and Hugo (1989). Mon. Weather Rev..

[17] Zhao Z, Chan PW, Wu N, Zhang JA, Hon KK (2020). Aircraft observations of turbulence characteristics in the tropical cyclone boundary layer. Bound. Layer Meteorol..

[18] Foken, T. *Micrometeorology*. 2 edn, 362 (Springer, 2008).

[19] Zhang F, Pu Z (2017). Effects of vertical eddy diffusivity parameterization on the evolution of landfalling hurricanes. J. Atmos. Sci..

[20] Tang J, Zhang JA, Aberson SD, Marks FD, Lei X (2018). Multilevel tower observations of vertical eddy diffusivity and mixing length in the tropical cyclone boundary layer during landfalls. J. Atmos. Sci..

[21] Ming J, Zhang JA (2018). Direct measurements of momentum flux and dissipative heating in the surface layer of tropical cyclones during landfalls. J. Geophys. Res. D Atmos..

[22] Lorsolo S, Schroeder JL, Dodge P, Marks F (2008). An observational study of hurricane boundary layer small-scale coherent structures. Mon. Weather Rev..

[23] Alford AA (2019). Near-surface maximum winds during the landfall of hurricane harvey. Geophys. Res. Lett..

[24] Alford AA (2020). Transition of the Hurricane boundary layer during the landfall of hurricane Irene (2011). J. Atmos. Sci..

[25] Choy CW, Lau DS, He Y (2020). Super typhoons Hato (1713) and Mangkhut (1822), part II: Challenges in forecasting and early warnings. Wthr.

[26] Hong S-Y, Noh Y, Dudhia J (2006). A new vertical diffusion package with an explicit treatment of entrainment processes. Mon. Weather Rev..

[27] Blackadar AK (1962). The vertical distribution of wind and turbulent exchange in a neutral atmosphere. J. Geophys. Res..

[28] Kepert JD (2012). Choosing a boundary layer parameterization for tropical cyclone modeling. Mon. Weather Rev..

[29] Powell MD, Vickery PJ, Reinhold TA (2003). Reduced drag coefficient for high wind speeds in tropical cyclones. Nature.

[30] Smith RK, Montgomery MT (2014). On the existence of the logarithmic surface layer in the inner core of hurricanes. Q. J. R. Meteorol. Soc..

[31] Stull RB (1988). An Introduction to Boundary Layer Meteorology.

[32] Pendergrass W, Arya SPS (1984). Dispersion in neutral boundary layer over a step change in surface roughness—I. Mean flow and turbulence structure. Atmos. Environ..

[33] Vickery PJ, Wadhera D, Powell MD (2009). A Hurricane Boundary Layer and Wind Field Model for Use in Engineering Applications. J. Applied Meteoro. Clim..

[34] Zhu P (2008). Simulation and parameterization of the turbulent transport in the hurricane boundary layer by large eddies. J. Geophys. Res..

[35] Kosiba KA, Wurman J (2014). Finescale dual-doppler analysis of hurricane boundary layer structures in Hurricane Frances (2004) at Landfall. Mon. Weather Rev..

